# Hysteretic Photochromic Switching (HPS) in Doubly Doped GaN(Mg):Eu—A Summary of Recent Results

**DOI:** 10.3390/ma11101800

**Published:** 2018-09-22

**Authors:** Paul R. Edwards, Kevin P. O’Donnell, Akhilesh K. Singh, Douglas Cameron, Katharina Lorenz, Mitsuo Yamaga, Jacob H. Leach, Menno J. Kappers, Michal Boćkowski

**Affiliations:** 1 SUPA Department of Physics, University of Strathclyde, 107 Rottenrow, Glasgow G4 0NG, UK; paul.edwards@strath.ac.uk (P.R.E.); akhilesh_singh343@yahoo.com (A.K.S.); douglas.cameron.2013@uni.strath.ac.uk (D.C.); 2 Alternative Energy Materials, CSIR-National Physical Laboratory, New Delhi-110012, India; 3 INESC/MN, IPFN, Instituto Superior Técnico, Universidade de Lisboa, Campus Tecnológico e Nuclear, Estrada Nacional 10, 2695-066 Bobadela LRS, Portugal; lorenz@ctn.tecnico.ulisboa.pt; 4 Department of Mathematical and Design Engineering, Gifu University, Gifu 501-1193, Japan; yamaga@gifu-u.ac.jp; 5 Kyma Technologies, 8829 Midway West Rd, Raleigh, NC 27612, USA; leach@kymatech.com; 6 Department of Materials Science and Metallurgy, University of Cambridge, 27 Charles Babbage Road, Cambridge CB3 0FS, UK; mjk30@cam.ac.uk; 7 Institute of High Pressure Physics PAS, Sokolowska 29/37, 01-142 Warsaw, Poland; bocian@unipress.waw.pl

**Keywords:** gallium nitride, rare earth ions, europium, photoluminescence, photochromism, qubit

## Abstract

Europium is the most-studied and least-well-understood rare earth ion (REI) dopant in GaN. While attempting to increase the efficiency of red GaN light-emitting diodes (LEDs) by implanting Eu^+^ into p-type GaN templates, the Strathclyde University group, in collaboration with IST Lisbon and Unipress Warsaw, discovered hysteretic photochromic switching (HPS) in the photoluminescence spectrum of doubly doped GaN(Mg):Eu. Our recent work, summarised in this contribution, has used time-, temperature- and light-induced changes in the Eu intra-4*f* shell emission spectrum to deduce the microscopic nature of the Mg-Eu defects that form in this material. As well as shedding light on the Mg acceptor in GaN, we propose a possible role for these emission centres in quantum information and computing.

## 1. Introduction

The doping of III-nitride semiconductors with rare earth ions, largely motivated by the promise of high-efficiency red GaN-based LEDs, has a fairly recent history [1]. By contrast, the *p*-type activation of GaN is well established, and underpins all commercial activities associated with the blue LED, although the nature of the Mg acceptor remains controversial.

In recent experiments, first described in [2], we implanted Eu as a ‘spectator ion’ to probe the lattice location of Mg in GaN(Mg):Eu [3]. We observed photochromic switching between different configurations of an Eu-Mg defect. By first cooling the sample in the dark and then using photoluminescence to monitor the process at a range of fixed temperatures, excitation densities and wavelengths, we managed to tune the characteristic switching time over many orders of magnitude. Linking the distinct Eu-Mg defect configurations with the shallow transient and deep ground states (STS and DGS) of the Mg acceptor, in the Lany–Zunger (L–Z) model [4], we determined an energy barrier of 27.7 meV for transitions between STS and DGS in agreement with theory. The experimental results suggest that at low temperatures holes are localized in deep ground states on N atoms axially bonded to Mg, as predicted by Lyons et al. [5]. 

The unexpected observation of transitions from excited ^5^D_1_ levels of Eu at lower temperatures completes our spectroscopic survey and explains an apparent anomaly in the temperature dependences of ^5^D_0_→^7^F*_J_* transitions [6]. Prolonged exposure of state-of the-art Eu-implanted HVPE GaN leads to photodissociation of the Eu-Mg centres when migrating Mg atoms fail to return, eventually leading to emergence of emission from un-associated Eu2 defects [7]. The spectroscopic effects of triple doping GaN(Mg):Eu with O or N are briefly described.

## 2. Materials and Methods

In the GaN lattice, Eu substitutes for Ga and takes the charge state of 3+ with 6 electrons in the *4f* shell. Hund’s rules predict a multi-electron ground state, labelled ^7^F_0_ in Russells–Saunders atomic term notation (*^2S+1^*L*_J_*), with spin and orbital components of 3ħ opposed to produce a total angular momentum *J* = 0. The states ^7^F_0,1,2,3_ form a close-lying ground state manifold connected to the ^5^D_0,1,2_ excited states by optical transitions of various types. (States lying higher than ^5^D_1_ are not considered here.) The atomic-like states are split further in the symmetric environment of the GaN crystal field, providing a valuable clue to the actual defect configurations, which may be reduced from the expected quasi-*C_3v_* symmetry of a substitutional site by the presence of intrinsic or extrinsic defects, resulting in what is called ‘site multiplicity’ [8]. For example, singlet levels with *J* = 0 do not split in any crystal field, while the *J* = 1 triplets split into doublet and singlet in an axial crystal field and into three separate levels in a non-axial field [9].

Different sites can sometimes be distinguished also by selective excitation [10,11]. For example, Eu2 defects, consisting of unassociated Eu atoms on Ga sites [11], can be excited only by light with photon energy greater than the GaN bandgap; Eu1 defects, associated with a vacancy [12], can be excited in addition by a broad band of photon energies just below the gap [10].

**Figure 1:**
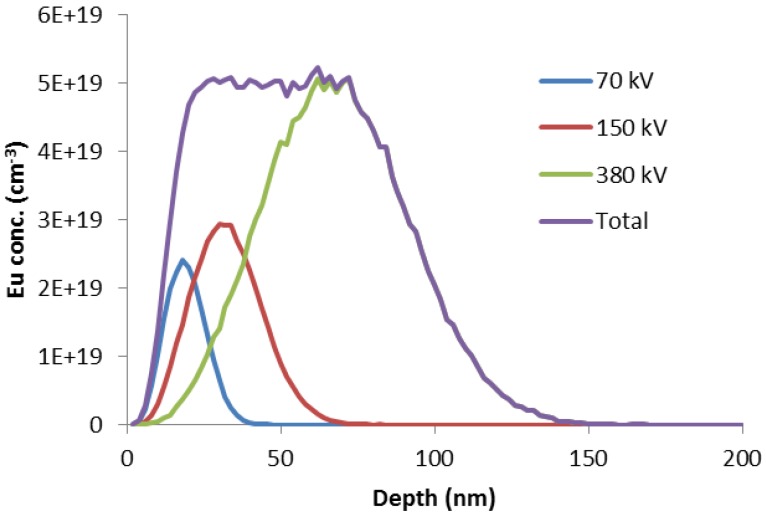
Ion implantation using multiple beam energies produces a top-hat dopant profile as shown in the above example, determined by Monte Carlo simulations using the SRIM code for Eu ions in GaN.

Doubly doping GaN to produce GaN(Mg):Eu samples proceeds in two stages. GaN doped with ~10^18^ to 10^20^ cm^−3^ Mg is grown at Cambridge by metal organic vapour phase epitaxy (MOVPE) as a 300 nm layer on an undoped 3 μm GaN template on 50 mm sapphire wafers. 1 × 1 cm^2^ offcuts are implanted by Eu ions in Lisbon to produce an approximately uniform depth profile, as shown in Figure 1. The lattice damage that results from ion implantation is annealed out under high pressure at high temperature in Warsaw (1400 °C, 1 GPa of N_2_). Details of the sample preparation can be found in Ref. [13]. One sample was grown and Mg-doped by HVPE prior to implantation/annealing.

Photoluminescence/excitation (PL/E) spectroscopy at Strathclyde is the main analytical technique used to characterize samples and examine HPS. Samples cooled in a helium cryorefrigerator are excited by monochromated light from a 1 kW Xe arc lamp, a 20 mW 355 nm laser or a pulsed nitrogen laser, in order of increasing achievable excitation density, analysed by a 2/3 m monochromator with a 1800 L/mm holographic grating and detected with a 16 bit 1064 × 128 pixel CCD camera.

## 3. Results

This section will be divided into the following subheadings. In Section 3.1, we present the spectrum of as-received GaN(Mg):Eu samples at room temperature, featuring mainly Eu0 luminescence, and introduce the 1-3-5 rule for Eu^3+^ defects in any host; in Section 3.2, we describe Hysteretic Photochromic Switching, as revealed in the temperature dependence of PL during a cooling-warming cycle; in Section 3.3, we describe the determination of the energy barrier that separates the photochromic defect configurations and link these observations to the Lany–Zunger model of the Mg acceptor; subsection Section 3.4 considers transitions from excited ^5^D_1_ states; Section 3.5 reveals the effects of irreversible photo-dissociation of Eu-Mg defects in GaN material of exceptional quality.

### 3.1. Eu0 Spectrum

**Figure 2:**
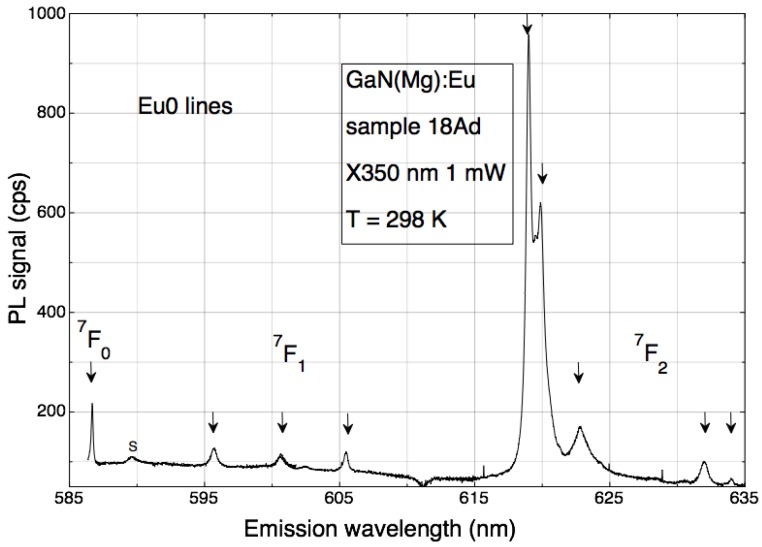
When excited with above-bandgap light at RT, spectrally pure GaN(Mg):Eu samples show only the Eu0 spectrum (reproduced from Ref. [9]).

At room temperature, the typical Eu0 PL spectrum clearly exemplifies the 1-3-5 rule. Transitions from the ^5^D_0_ excited state are identified in Figure 2 by the label of the terminal state, with degeneracies of 1 (^7^F_0_), 3 (^7^F_1_) and 5 (^7^F_2_), respectively. All degeneracies are lifted by a crystal field with lower than axial symmetry, to produce a set of well separated lines indicated by arrows.

The Eu0 spectrum appears only in Eu- and Mg- (double) doped GaN [1,2] and careful doping comparisons show that its maximum intensity is achieved when the concentrations of Eu and Mg are approximately equal. This leads us to a model of the Eu0 defect comprising a single Eu atom in close association with a Mg atom, both substituting on the Ga sub-lattice.

### 3.2. HPS

When samples are cooled below about 50 K, we encounter (with much surprise) the photochromic switching of the Eu0 spectrum, in all of its components, to a completely different one.

**Figure 3:**
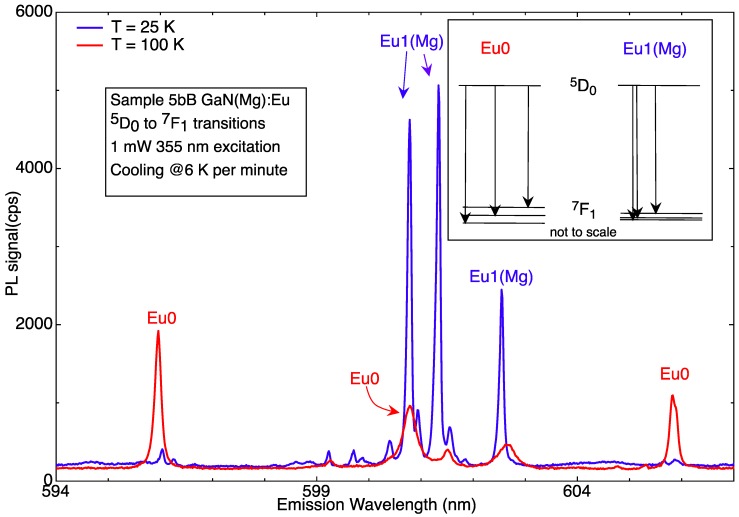
Showing the two very different ^5^D_0_ to ^7^F_1_ spectral patterns for the photochromic pair Eu0 and Eu1(Mg), dominant at 100 K and 25 K respectively (reproduced from Ref. [9]).

We assigned the low-temperature spectrum to Eu1(Mg), due to its superficial spectral similarities with the Eu1 centre observed in undoped GaN:Eu. Eu1(Mg) is much more symmetrical than Eu0, with, most notably, the ^7^F_1_ state splitting as 3 = 2 + 1, that is to say into a (close-lying) doublet and a singlet [8]. The profound alteration of the spectrum between 100 K and 25 K is shown in Figure 3.

**Figure 4:**
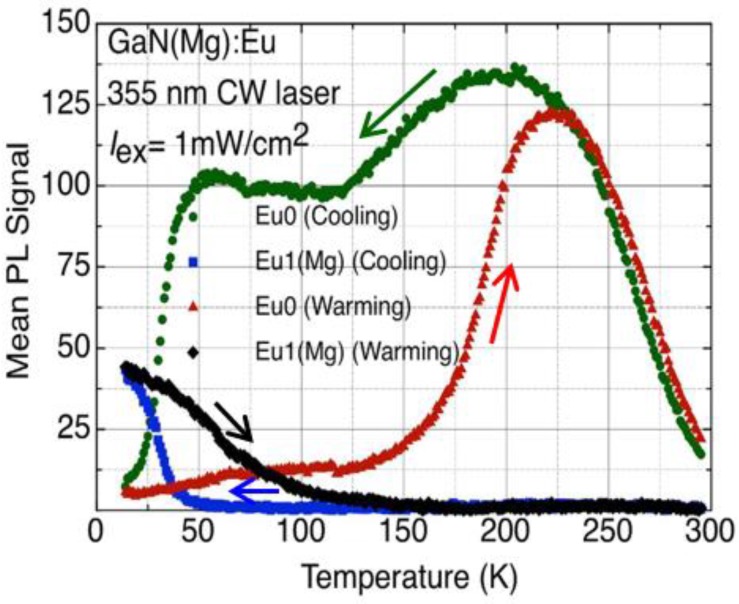
Showing various hysteresis loops associated with the HPS of Eu0-Eu1(Mg) during a cooling-warming cycle. The mean signal intensities are plotted against temperature for cooling and warming runs, as indicated by arrows, undertaken at a fixed rate of 6 K per minute. Data are recorded at 1 K intervals (reproduced from Ref. [3]).

The switching of Eu0 to Eu1(Mg) is soon found to be hysteretic (hence *H*PS): the reverse process occurs at a much higher temperature. The different hysteretic behaviours of Eu0 and Eu1(Mg) are shown in Figure 4 for a typical experimental cycle.

Switching upon cooling (switchdown) offers the clearest perspective: the intensity of Eu0 drops to zero over a small temperature range, while that of Eu1(Mg) increases to a maximum value at the base temperature of our cryostat, ~10 K. 

Switchback from Eu1(Mg) to Eu0 upon sample warming is more complicated and not fully understood. The maximum Eu1(Mg) signal at 10 K does not equal the maximum Eu0, but we ascribe this difference to one of transition probability between the more symmetric centre (less probable for intra-*f* shell transitions) and the less symmetric one. We conclude that the switching is one-to-one with respect to defect numbers. The anomalous decrease in intensity of the Eu0 signal between its peak at 200 K and the start of the plateau at 125 K, explained only recently [6], will feature in Section 3.4.

### 3.3. Quantifying Photochromism

Without a mathematical model of the temperature dependence, it is difficult to extract physical parameters from the experiments described in Section 3.2. With the realisation that the observation of photochromism requires light both to *observe* the effect, through PL, and to cause it, we devised a measurement protocol in which the sample was cooled to a fixed temperature in the dark and then subjected to a fixed wavelength and intensity of light for periods long enough that switching could be called essentially complete, while measuring the spectrum at 0.1 or 0.25 s intervals. (For brevity—and to reflect the situation in which the Mg-Eu complex is ‘surprised’ out of its non-equilibrium state by the sudden illumination—we familiarly refer to this as the ‘Ninja!’ protocol.)

Some general results can be cited for these experiments. For fixed excitation power and wavelength, the switching profile is hyperbolic in time, with a characteristic time constant *τ* that can be extracted from the temporal dependence of switching by fitting:

*I*(*t*)/*I*(0) = 1/(1+ *t*/ *τ*) (1)

to the dataset, where *I*(*t*) is the (mean) PL intensity of the unswitched (Eu0) component at time *t*. The switching rate slows with increasing temperature, in an anti-Arrhenius relationship that reflects the fact that colder samples are further from equilibrium; it is the return to equilibrium that is captured in the Ninja! runs. Increasing the incident power decreases the switching time, as might be expected.

**Figure 5:**
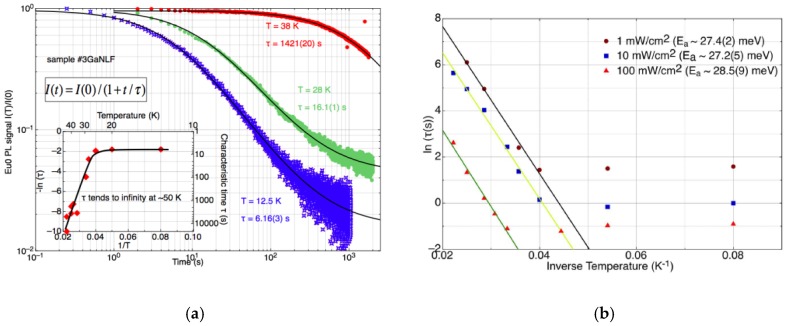
(**a**) Temperature dependence of switching at fixed excitation density and wavelength, the inset showing temperature independence below 20 K; (**b**) anti-Arrhenius fits to the temperature dependence above ~20 K show an independence of the activation energy on the power density (after Ref. [3]).

Some example results for a particular sample are illustrated in Figure 5a,b. By fitting the temperature dependence of the switching time, the barrier between the photochromic states is estimated to be 27.7 meV, more or less independent of the excitation power density.

### 3.4. Transitions from ^5^D_1_ Levels

In conventional PL of semiconductors, it is expected that thermalisation between excited states should decrease the relative intensity of transitions originating on states lying higher in energy as the temperature decreases. The intensity should mirror a decreasing population of the higher lying levels through the operation of Boltzmann statistics.

**Figure 6:**
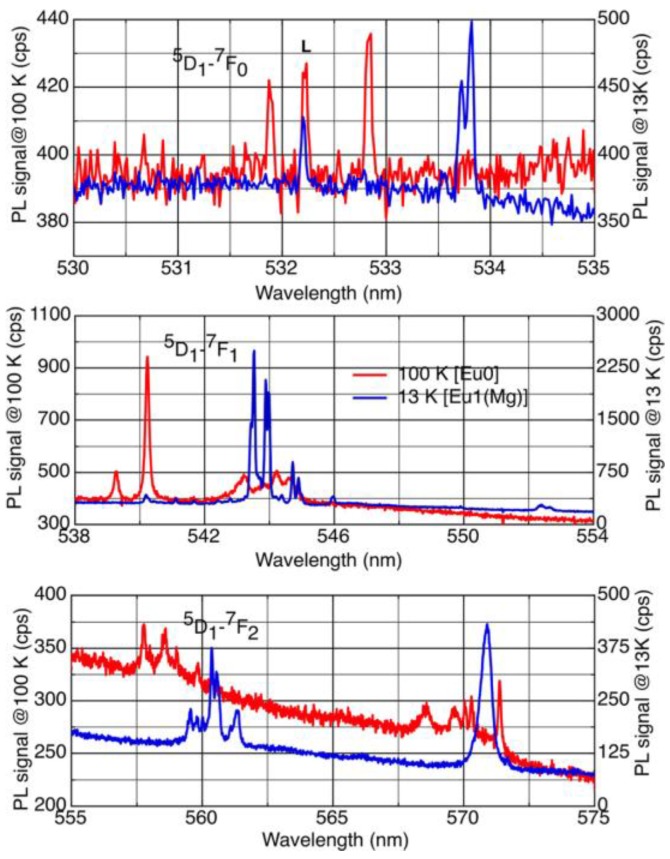
The transitions from the ^5^D_1_ states of both Eu0 and Eu1(Mg) configurations of the Eu-Mg defects can be observed at the appropriate temperatures, allowing for photochromic switching (reproduced from Ref. [6]). For an explanation, see text.

**Figure 7:**
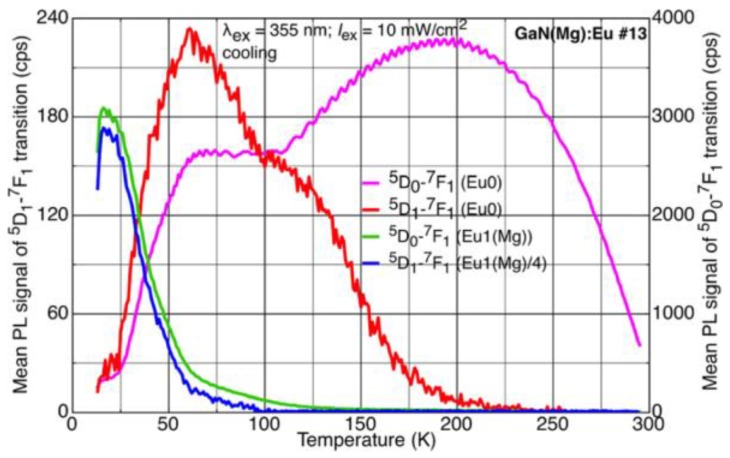
Temperature dependences of intensities of transitions as indicated (cooling run). Note the anti-correlation of Eu0 PL signals from the ^5^D_1_ and ^5^D_0_ states in the temperature range 200 K to 100 K (reproduced from Ref. [6]).

On the contrary, transitions from the ^5^D_1_ levels in GaN(Mg):Eu are stronger at lower temperatures. Such transitions for both configurations of Eu-Mg defects are shown in Figure 6, and their temperature dependences in Figure 7.

The ^7^F_0,1,2_ level splittings derived from an analysis of the spectra of Figure 6 confirm those found previously for the stronger transitions from the ^5^D_0_ states to these levels. This allows us to determine the ^5^D_1_ to ^5^D_0_ energy separation as well as the (relatively small) splittings within the ^5^D_1_ levels caused by the crystal field [6].

### 3.5. Photodissociation

When samples are subjected to prolonged exposure to above-bandgap light at low temperatures, further spectral changes occur, which we refer to as ‘second switching’ or ‘blitching’: bleaching of the total emission is accompanied by switching from Eu1(Mg) to a set of ever more symmetrical centres, labelled Eu1(Mg2), Eu1(Mg3) etc. [14,15,16]. 

In MOVPE-grown samples, second switching occurs at a leisurely pace, necessitating experimental runs as long as 36,000 s to establish clear trends. However, for a state-of-the-art free-standing GaN(Mg) sample prepared by HVPE at Kyma Technologies, and doped with Eu as described in Section 2, the blitching process is rapid, and ultimately destructive [7].

**Figure 8:**
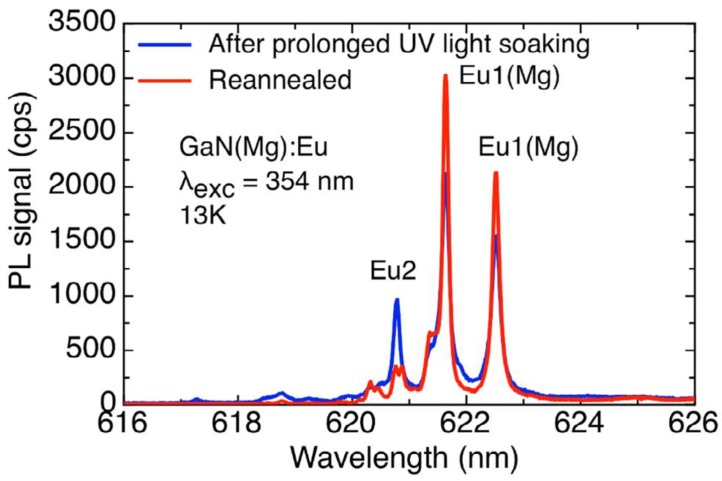
After prolonged exposure to UV light at low temperature, this ‘half-cooked’ sample displays the characteristic 620.9 nm line of the prime Eu2 centre, produced by unassociated substitutional Eu^3+^ ions [11].

Figure 8 shows the eventual result of such an experiment. Following a first set of runs that resulted in irreversible spectral alterations, the sample was re-annealed at high temperature and pressure, but only partly recovered its original spectral purity. (A sample that has lost the Eu0 dominance of the RT spectrum, shown in Figure 2, is said to be ‘cooked’.)

## 4. Discussion

When taken together, the various pieces of experimental evidence summarised in Section 3 lead us to form a precise microscopic model of the Mg-Eu complexes that form in p-type GaN.

We know from observing maximum Eu0 emission when Mg and Eu concentrations match that the centre is likely to involve a single Eu atom in close association with a single Mg atom. Both Mg^2+^ and Eu^3+^ will favour cationic sites in the GaN lattice, so we propose that they both substitute for Ga and share a common nitrogen nearest neighbour.

We next consider the change in symmetry during the switch from Eu0 to Eu1(Mg) on cooling the sample (switchdown). This indicates that a local distortion in the lattice is induced during cooling, a kind of phase change occurring on the nanoscale, and shows the Eu^3+^ ion to be in a higher symmetry environment in the Eu1(Mg) state.

A further clue regarding the nature of this distortion comes from the activation energy obtained from quantifying the light-induced switching of Eu0 to Eu1(Mg) at low T: this value (27.7 meV) is close to the barrier height predicted to exist between the two Mg acceptor states proposed by Lany and Zunger as the ‘curious case of the shallow Mg_Ga_ deep state’ [4]. Hybrid density functional calculations predicted formation of a shallow transient state, effective in the p-type activation, and a deeper ground state; further calculations by Lyons et al. [5] supported the existence of such a deep state, specifically attributing it to localization of a hole at an axial N atom.

We proposed [9] that the switching between Eu0 and Eu1(Mg) is the spectroscopic signature of the transition between these two Mg acceptor states, as observed by the nearby Eu ‘spectator’ atom. Localization of a hole on the magnesium’s axial N bond (i.e., the deep ground state) is the stable configuration at low temperature, giving Eu1(Mg); delocalization of the hole at higher temperatures, and the resultant local lattice distortion, results in a change in the crystal field splitting and the switch to Eu0. 

**Figure 9:**
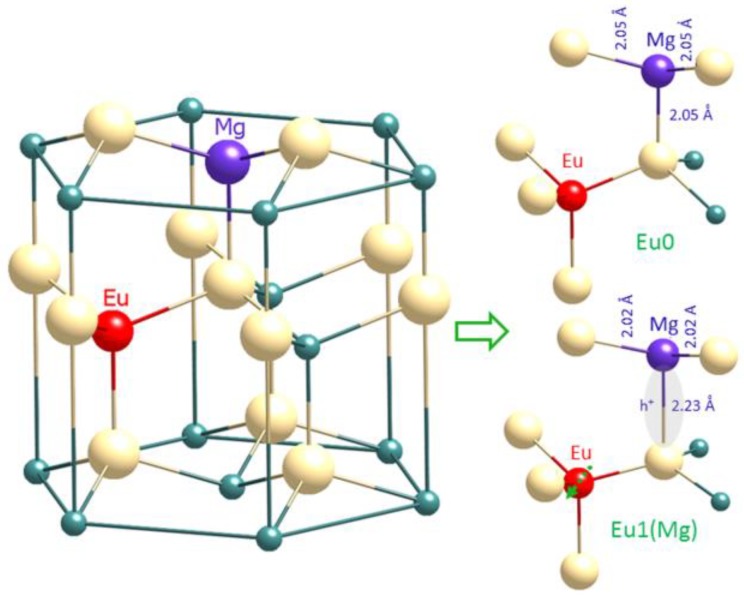
Proposed microscopic model showing Eu sharing a nitrogen atom axially bonded to the Mg. The distortion of this arrangement due to the localization of a hole at this bond is also depicted (reproduced from Ref. [3]).

Our microscopic model is sketched in Figure 9.

One effect of the prolonged UV exposure of these materials is the emergence of additional centres whose line splittings reveal successively increased symmetry [15]. This indicates that a fraction of Mg atoms has *physically migrated* to lattice sites away from their Eu partner. This eventually leads to the appearance of the Eu2 centre, identified as the ‘prime’ defect in unimplanted GaN:Eu (i.e., a simple substitutional Eu atom on a cation site); the Mg and Eu must now be sufficiently separated to be entirely disassociated.

Finally, we have made recent observations (to be fully reported at a later date) of the effects of co-implanting the GaN:Mg(Eu) with additional anion species to give triply-doped material. We find that the introduction of excess N atoms enhances Eu0 production and reduces the intensity of vestigial Eu1 lines, supporting Mitchell et al.’s previous identification of this centre as substitutional Eu associated with a nitrogen vacancy [12]. The introduction of O results in a new centre, with the same symmetry as Eu0, but not exhibiting the switching behaviour. We interpret this as being due to the formation of an Eu-O-Mg defect complex in which the strong Mg-O bond disrupts the ability of a hole to localise in the deep ground state which is central to the Eu0-Eu1(Mg) switching.

## 5. Conclusions

The spectroscopy of europium ions in GaN shows behaviour which is complex due to site multiplicity—and made dramatically more so by the presence of Mg. The variation of the spectra with factors such as time, temperature, illumination and co-doping allow us to probe the mean local environment of an ensemble of Eu^3+^ ions, and to use these as ‘spectators’ to directly observe previously theorised acceptor states of the commercially important Mg dopant in GaN. Continuing work will aim to extend this to observing individual Eu^3+^ ions in microscope-based versions of the experiments reported in Section 3. This work will allow us to investigate the possibility of applications of REI-implanted semiconductors that go beyond the LED. We have identified in this defect a 2-level system which can be switched and read optically; learning to manipulate these states with more precise control has the potential to open up a new branch of solotronics (we propose the name ‘QuantREIonics’) aimed at quantum information and computing.
